# Academic career intentions in the life sciences: Can research self-efficacy beliefs explain low numbers of aspiring physician and female scientists?

**DOI:** 10.1371/journal.pone.0184543

**Published:** 2017-09-14

**Authors:** Nurith Epstein, Martin R. Fischer

**Affiliations:** Institut für Didaktik und Ausbildungsforschung in der Medizin, Klinikum der Universität München, Munich, Germany; Kyoto University, JAPAN

## Abstract

A lack of physician scientists as well as a high female dropout rate from academic medicine and basic life sciences is a concern in many countries. The current study analyzes academic career intentions within a sample of recent doctoral graduates from medicine and basic life sciences (N = 1109), focusing on research self-efficacy beliefs as explanatory variable of gender and disciplinary differences. To ensure that differences in research self-efficacy could not be attributed solely to objective scientific performance, we controlled for number of publications and dissertation grade. The results of multivariate analyses pointed to a strong and significant association between research self-efficacy and academic career intentions (ß = 0.49, p<0.001). The lower academic career intentions of medical doctoral graduates were no longer significant when controlling for research self-efficacy. Within the field of medicine, female doctoral graduates expressed lower research self-efficacy beliefs and academic career intentions. When controlling for research self-efficacy, the correlation between gender and academic career intention was no longer significant. In contrast, no gender differences were found within the basic life sciences with respect to neither academic career intentions nor research self-efficacy.

## Introduction

As with other countries, a lack of physician scientists [1–7]), as well as female scientists in medicine and basic life sciences in particular [8–10], is of concern in Germany. Low numbers of medical graduates pursuing academic research careers constitute a serious problem since interdisciplinary research in the life sciences is important to generate new knowledge [11, 12]. The participation of physician scientists is specifically important for enabling the translation from “bench to bedside” [13–15]. Since female students make up over half of the student population in both medicine and basic life sciences, the high female scientist dropout rate [16–18] needs further insight [18].

Both in medicine and in basic life sciences, the PhD is considered the standard degree in Germany [5, 19, 20]. While in both fields the PhD is the formal validation of the ability to perform research independently, there are some main differences in procedure, purpose and job market circumstances: In basic life sciences, the PhD usually succeeds the master’s degree and takes around 4,5 years [21]. It is necessary for pursuing a career in academic research and is also recommended for careers outside of academia. Due to an overproduction of (PhD) graduates in basic life sciences, there are not enough available jobs in and outside of academia [22–24]. PhD graduates in basic life sciences might be pushed into continuing as a postdoc due to limited career prospects outside of academia [24].

Medical students usually start the PhD (Dr. med.) during undergraduate studies and the dissertation represents their first scientific thesis. Like the PhD, the Dr. med. is a sufficient precondition for pursuing an academic research career. In comparison to basic life sciences, the doctoral degree is usually attained within a shorter time frame and has been criticized in terms of its scientific quality [25, 26]. Although the degree is not required for practicing as a physician, the doctoral degree has become so common, that it is closely associated with the profession of a physician as such. Physicians may pursue the Dr. med. because they worry about career consequences (perception of employers and patients). A professional doctorate, like the MD in other countries, does not exist in Germany (yet). In contrast to graduates from basic life sciences, there is a high demand for physicians and they face excellent career opportunities as clinicians [1, 3, 5].

When pursuing an academic research career in Germany, doctoral graduates from medicine and basic life sciences face similar circumstances. While academic research is a career path with an uncertain outcome in many countries, this uncertainty is particularly high in Germany [27]. A permanent position in academic research is almost exclusively possible with the attainment of a full professorship around six years after the doctorate. As a result, German academia is also referred to as a system of “up or out” [28]. Full professorships only account for around 10 percent of academic research staff and are usually appointed at an age around 40 [29]. Hence, the German system does not make room for lateral entrants and an early completion of the doctorate is required to acquire a position long term.

While there are no data directly comparing academic career pursuit between medicine and other basic life sciences, a study of Briedis and colleagues [30] showed that German medical doctoral graduates were 43 percent less likely to stay in academia in comparison to doctoral graduates from math and natural sciences. Whereas the reference group (math and natural sciences) also includes non-life sciences fields, this study provides a first indicator of a lower academic career pursuit of medical doctoral graduates.

Against this backdrop, the current article explores differences in academic career intentions between doctoral graduates from medicine and basic life sciences focusing on research self-efficacy as explanatory variable. Research self-efficacy beliefs designate a person’s beliefs to perform research related tasks successfully in the future [31, 32]. Since objective performance alone does not guarantee long term career success in academic research (acquiring a permanent position/professorship), research self-efficacy beliefs may pose a meaningful influence on academic career intentions as some empirical evidence suggests [18, 31, 33, 34]. While physician scientists can circumvent the risky “academic pipeline” by combining research with clinical practice, research self-efficacy beliefs could be an important explanatory factor for low numbers of aspiring physician scientists, since doctoral graduates from medicine are less experienced in conducting research in comparison to those from basic life sciences. Medical students may not feel prepared to continue their research careers.

Research self-efficacy beliefs could also provide an explanation for a higher female academic career dropout: several studies have suggested that females hold lower research self-efficacy beliefs [14, 18, 31, 32, 34, 35]. A limitation to most previous studies, however, is the lack of control for actual scientific performance (e.g. publications, doctoral thesis grade) [32]. It can be concluded, that research self-efficacy beliefs may be a contributing factor to the higher amounts of female drop outs in the fields of medicine and basic life sciences within the postdoc phase, but the empirical evidence is not sufficient to support that claim.

Hence, the current article aims to explore the role of research self-efficacy in academic career intentions of medical and basic life sciences doctoral graduates and related gender differences. The article is structured as follows: first, an overview of the research literature on the effects of research self-efficacy and gender differences in research self-efficacy is provided. The derived hypotheses are subsequently tested on a sample of medical and basic life sciences doctoral graduates from Germany. In the last sections, the results and practical implications are discussed.

## Background and hypotheses

### Research self-efficacy beliefs in the academic career context: Previous findings

Self-efficacy beliefs (short: self-efficacy) designate one’s beliefs to successfully conduct specific courses of actions in the future [36, 37]. Empirical studies in the educational field find self-efficacy beliefs to be positively correlated with achievement motivation, persistence [38, 39] and academic performance [40–43]. Referring to the relevance of self-efficacy within the career/occupational context, studies show correlations with career intentions, for example choice of occupational area [44] but also career success/performance [45–47].

Within the context of academic research, empirical studies have shown that research self-efficacy beliefs are positively related to the intention to pursue an academic research career [18, 31, 33, 34, 48], and the intention to study for a PhD [34]. A limitation of most of the aforementioned studies is that they do not control for objective levels of scientific performance, such as the number of published articles. As Jöstl and colleagues [32] note “[a]nother aspect which should be examined in future studies concerns more detailed information about the sample of doctoral students, namely their dissertation progress and their actual abilities” (ibid., p. 117). An exception is the study by Gibbs and colleagues [18], whose results, however, may be influenced by their heterogeneous group of participants, who were recruited from diverse channels. Participants had obtained their PhD a couple of years prior to the study. Hence, different career experiences after the PhD may have had a stronger influence on participants’ research self-efficacy beliefs than their experiences as a PhD student. Thus, conclusions about the predictive power of research self-efficacy beliefs beyond actual objective scientific performance cannot be derived with certainty. Against the empirical evidence about (research) self-efficacy and its relation to achievement motivation, it is hypothesized that research self-efficacy beliefs are significantly related to academic career intentions when controlling for objective scientific performance:

Hypothesis 1 Research self-efficacy beliefs are, beyond objective scientific performance (publications, grade of the doctorate), significantly related to academic career intentions in doctoral graduates from medicine and basic life sciences.

Due to the averagely lower research experience of medical doctoral graduates, it is hypothesized that medical doctoral graduates express lower research self-efficacy beliefs in comparison to basic life sciences doctoral graduates:

Hypothesis 2 Medical doctoral graduates express significantly lower academic career intentions in comparison to doctoral graduates from other life sciences.

It is further hypothesized that, when controlling for research self-efficacy, medical doctoral graduates and life sciences doctoral graduates do not significantly differ with respect to their academic career intentions:

Hypothesis 3 Research self-efficacy beliefs mediate the effect of field of study on academic career intention: under control of research self-efficacy, medical and basic life sciences doctoral graduates do not significantly differ with respect to their academic career intentions.

### Gender differences in research self-efficacy

Despite a high percentage of female doctoral graduates from medicine and basic life sciences, the female dropout within the postdoc phase through to professorship is very high, resulting in a below average percentage of female professors [8–10]. While 20 percent of full professorships in Germany are held by women [49], the percentage of female professorships in both medicine and basic life sciences is close to 10 percent [8, 9]. This pattern is not unique to Germany, as it is internationally observed [9, 10, 18, 50, 51]. In addition to the general relationship between research self-efficacy and academic career intentions, several studies report lower research self-efficacy beliefs by female (doctoral) graduates and researchers [14, 31, 32, 34, 52]. Spies and Schute [34] find that females in mathematics and biology have significantly lower self-efficacy beliefs and intentions to pursue a doctorate. Their results also supported self-efficacy as a possible mediator between gender and the intention to pursue a doctorate. An additional interesting result was that gender differences in mathematics (male dominated field of study) were larger in comparison to the field of biology (female dominated field of study).

Within a clinical-research training program, Bakken et al. [14] found significantly lower research self-efficacy beliefs in female physicians in comparison to their male counterparts. These effects appeared before and after training. The previously mentioned study by Gibbs and colleagues [18] found significant gender differences in research self-efficacy among biomedical PhDs. In addition, gender differences in academic career intentions stayed significant when controlling for research self-efficacy. Berweger and Keller [31] found lower female research self-efficacy beliefs and academic career intentions in a sample of doctoral students from the fields of philosophy, human and social sciences. However, the separate analysis of female and male doctoral students limits the insights on research self-efficacy as a possible mediator between gender and academic career intentions.

Consistent with these results Jöstl et al. [32] also found lower female research self-efficacy beliefs within a sample of doctoral students from various, often unrelated fields. Hemmings and Kay [52], who surveyed full-time lecturing staff from various fields with and without doctoral degree, found lower efficacy beliefs in females with respect to “performing tasks that pertained to writing major works and reviewing articles/books as well as undertaking professional engagement activities” (ibid., p. 249). While lower research self-efficacy beliefs expressed by females seem to be a plausible explanation for the underrepresentation of women in academic research, some studies do not find any gender differences in self-efficacy [48] or related constructs [53].

Inconsistent findings could be a result of different fields of study being analyzed, since previous results suggest gender differences to be domain specific [34, 54]. Most studies on the topic analyze various fields of study conjointly. Considering fields of study separately might be important since fields vary in their gender ratios for both the student population but also academic staff. Gender ratios in higher positions may convey information to junior scientists of their own chances to survive the academic career pipeline [55].

Variations in used instruments may be another factor contributing to inconsistent findings. Some scales only focus on the research process itself [48, 56, 57] and neglect other important activities in academia, such as networking, acquiring third party funds etc. [58]. However, the simplest answer to the inconsistent findings may be unobserved heterogeneity: whereby, actual differences in scientific performance may possibly explain lower levels of research self-efficacy expressed by females. This is an argument which is also supported by several findings suggesting that female researchers publish less [59–63] despite some evidence that this trend might fade or even reverse in younger generations [64]. Following from this, it is hypothesized that there are no gender differences in research self-efficacy and academic career intentions when controlling for objective scientific performance:

Hypothesis 4 When controlling for measures of objective scientific performance, there are no significant gender differences in research self-efficacy beliefs.
Hypothesis 5 When controlling for measures of objective scientific performance, there are no significant gender differences in the intention to pursue an academic research career.

## Method

The study was approved by the ethical committee of the Klinikum der Universität München (http://www.med.uni-muenchen.de/forschung/ethik/index.html)

### Sample

To investigate the hypothesized relationship, data of an online survey was used [65, 66]. The survey contains data of medical and basic life sciences doctoral graduates who graduated between 2013 and 2015. Doctoral graduates were recruited from medical and biological faculties of 13 German universities. Participants received their doctoral degree within one year prior to the study. Doctoral graduates older than 40 years (N = 80) were excluded from the analysis, due to the limited possibilities to pursue an academic research career when completing the doctorate at that age in Germany (cf. Introduction). Further, smaller fractions of graduates from other fields (social sciences and humanities, N = 36, dental medicine, N = 113) were not considered due to small sample sizes and issues of representativeness. The final sample comprised 1,109 doctoral graduates, 538 medical doctoral graduates and 571 life sciences doctoral graduates. As expected, the majority of the respondents were female: 63 percent in medicine and 60 percent in life sciences. This gender ratio appears to be representative for those fields of study [16, 17, 67]. Moreover, the respondents’ age (mean = 31) did not significantly differ. Medical doctoral graduates reported an average doctoral study duration of 69 months, while doctoral graduates from basic life sciences reported 55 months. While empirical evidence suggest that the duration of a medical PhD is shorter on average [68], the study asked for the date of beginning and completion of the doctorate. Since medical students often start their doctorate within their regular course of studies, they cannot work on the dissertation project full-time and probably encounter longer phases in which they don’t actually work on their thesis.

### Measures

#### Research self-efficacy

A domain-specific, nine item research self-efficacy scale was developed. The scale assesses the respondent’s confidence with respect to successfully mastering important tasks of an academic research career (e.g. “Now that I have my PhD, I am sure I can frequently publish research findings in journals with peer review process”, complete list of items in S1 Appendix). The items were in part adapted from the scale by Berweger and colleagues [31, 69], additional central activities of an academic research career [58], such as attracting third party funding, were added. Items were assessed on 5-point-Likert scales (1 = completely disagree; 5 = completely agree) and summarized as an additive index, scaled from 1 to 5. The reliability of the scale was very good (Cronbach’s alpha: 0.93).

#### Academic career intentions

Inspired by Berweger and Keller’s study [31] academic career intentions were operationalized as expressed long term career goals. Respondents were asked on a 5-point Likert scale (1 = low aspirations; 5 = high aspirations), if in the long run, they would 1) aspire for an academic research career and 2) aspire for a professorship. The two items were summarized as an additive index, scaled from 1 to 5. The reliability of the scale was very good with a Cronbach’s alpha of 0.92.

#### Performance accomplishments

As noted earlier, performance accomplishments are important sources of self-efficacy [36] and, hence, are important control variables. Since publications, as the best predictor of achieving full professorship [70], seem to be the most important performance indicator in academia, the number of articles published during the doctorate as first and co-author were assessed. Furthermore, the attained grade of the doctorate was considered and operationalized as categorical variable (summa cum laude (excellent), magna cum laude (very good), cum laude (good) or lower, and no grade received). In addition, the duration of the doctorate was included as a performance variable since participants’ research self-efficacy could be lowered as a result of needing an above average amount of time to complete their doctoral research study.

For medical doctoral graduates only, a binary variable for having conducted an experimental or non-experimental doctorate was included. Experimental dissertations (i.e. in vivo or in vitro biomedical research) in medicine are considered the most demanding from a scientific point of view [2], having conducted an experimental doctoral research study should be related to higher research related self-efficacy.

#### Work experience

In addition to performance, we controlled for national and international conference contributions. While conference contributions do not have the importance of publications, they are an important part of academic research careers and represent experience in interacting with the scientific community. Being accepted at conferences might, specifically to doctoral students with little experience, boost confidence in their research capabilities / research self-efficacy. Moreover, we controlled whether the doctoral graduates were part of a working group during the time of conducting their dissertation (binary variable). Being a part of a working group, hence being in contact with other more experienced researchers, could affect research competencies and knowledge beyond the specific dissertation project, thereby affecting research self-efficacy as well.

### Data analysis

Analyses were carried out with the statistical package Stata, Release 12 [71]. In the multivariate analyses missing values were imputed with full information maximum likelihood estimation (FIML). The amount of missing values for all items did not exceed 15 percent. To analyze the associations between disciplines, research self-efficacy and academic career intentions, path analysis was employed [72]. Path analysis as an “extension of multiple regression […] can examine situations in which there are several final dependent variables and those in which there are "chains" of influence, in that variable A influences variable B, which in turn affects variable C” (ibid, p.115). In the path analyses, correlations between variables and academic career intentions that are statistically mediated by research self-efficacy are designated as “indirect effects”. Hence, those variables are significantly related to academic career intentions through their correlation with research self-efficacy, in contrast to variables that have a “direct effect” on academic career intentions, irrespective of their correlation with research self-efficacy. The term effect should not be comprehended as implying causality, but is the usual nomenclature in path analysis (ibid).

## Results

### Descriptive results and bivariate comparisons

Before conducting multivariate analyses and hypotheses testing, descriptive and bivariate analyses were conducted to explore variable distributions, field, and gender differences in the dependent and independent variables. Table 1 shows the mean differences for male and female doctoral graduates for the fields of medicine and life sciences. Field and gender differences were apparent in performance variables in that medical doctoral graduates had published fewer first and co-author articles, and that female doctoral graduates had published significantly fewer articles as a first and co-author in both medicine and life sciences. With respect to conference contributions, medical graduates attended fewer national and international conferences. Female graduates in both fields attended only fewer international conferences.

**Table 1:** Model variables by field of study and gender.

	Male			Female				
**Field of Study: Medicine**	**M**	**SD**	**N**	**M**	**SD**	**N**	**p**	**d**
**Dependent Variables**								
**Academic Career Intention**	2.18	1.30	177	1.74	1.30	306	0.000	0.45
**Research Self-Efficacy**	2.82	0.96	177	2.38	0.90	289	0.000	0.44
**Independent Variables**								
**1st Author Publications**	0.56	0.75	174	0.32	0.66	283	0.000	0.24
**Co-Author Publications**	1.01	1.20	167	0.87	1.06	301	0.109	0.13
**International Conferences**	0.56	1.33	183	0.36	1.25	310	0.092	0.20
**National Conferences**	0.98	1.58	192	0.76	1.54	327	0.058	0.22
**Field of Study: Life Sciences**	M	SD	N	M	SD	N	p	d
**Dependent Variables**								
**Academic Career Intention**	2.46	1.40	180	2.28	1.31	275	0.081	0.18
**Research Self-Efficacy**	3.66	0.69	173	3.59	0.71	253	0.142	0.07
**Independent Variables**								
**1st Author Publications**	1.89	1.73	182	1.33	1.29	280	0.000	0.57
**Co-Author Publications**	2.21	1.88	180	1.92	1.82	273	0.056	0.29
**International Conferences**	2.80	2.60	196	2.2	2.1	301	0.013	0.56
**National Conferences**	2.96	2.28	199	2.82	1.99	302	0.456	0.14

Note: Means (M) and standard deviations (SD) are rounded to the second decimal place; p-values are rounded to the third decimal place, N = number of doctoral graduates, d = effect size/mean difference.

Further, medical graduates were less frequently associated with a working group (35 percent vs. 60 percent); however, there were no gender differences in this regard. With respect to achieved grade, 17 percent of life sciences doctoral graduates received a grade of summa cum laude opposed to 8 percent in medicine. In addition, male life sciences doctoral graduates more frequently achieved a grade of summa cum laude then their female counterparts (21 percent vs. 11 percent). This was also true for medicine: 9 percent of male medical graduates received a grade of summa cum laude opposed to 6 percent of females. Male medical graduates also conducted an experimental dissertation slightly more frequently than female medical graduates (46 percent vs. 40 percent).

Regarding the dependent variables of interest (research self-efficacy and academic career aspirations), medical graduates expressed lower research self-efficacy beliefs and academic career aspirations. On these variables, no gender differences were apparent in the life sciences. However, female medical graduates expressed significantly lower research self-efficacy beliefs and lower intentions to pursue an academic research career in comparison to their male counterparts.

### Multivariate analyses

#### Field of study, research self-efficacy and academic career intentions

To test hypothesized differences between medical and life sciences doctoral graduates (Hypotheses 1–3), a path analysis was carried-out with research self-efficacy as mediator variable and academic career intentions as dependent variable. The results of the path analyses are depicted in Table 2 In support of Hypothesis 1 research self-efficacy beliefs were significantly related to academic career intentions while controlling for objective performance measures. Furthermore, in support of Hypothesis 2, medical doctoral graduates expressed significantly lower research self-efficacy beliefs and academic career intentions. In support of Hypothesis 3, differences in academic career intentions were no longer significant when controlling for research self-efficacy.

**Table 2:** Path analysis—Field differences in research self-efficacy (RSE) and academic career intentions (ACI).

Independent Variables	Model 1: Direct Effects on RSE			Model 2: Direct Effects on ACI			Model 3: Indirect Effects on ACI		
	ß	SE	p	ß	SE	p	ß	SE	p
**Research Self-Efficacy**				0.49	0.03	0.000			
**Field of Study: Medicine**	-0.24	0.03	0.000	0.21	0.04	0.000	-0.12	0.05	0.000
**Grade: summa cum laude (excellent)**	0.17	0.03	0.000	0.20	0.04	0.000	0.08	0.07	0.000
**Grade: magna cum laude (very good)**	0.11	0.03	0.001	0.05	0.04	0.180	0.05	0.04	0.002
**No grade**	0.04	0.03	0.162	0.00	0.03	0.921	0.02	0.09	0.165
**(reference category: cum laude (good) and lower)**									
**Publications: 1st author**	0.20	0.03	0.000	0.05	0.04	0.179	0.10	0.02	0.000
**Publications: Co-Author**	0.02	0.03	0.520	0.04	0.03	0.166	0.01	0.01	0.521
**Duration of Doctorate**	-0.07	0.03	0.005	-0.02	0.03	0.464	-0.04	0.00	0.007
**National Conferences**	0.09	0.03	0.004	-0.09	0.03	0.007	0.04	0.01	0.004
**International Conferences**	0.12	0.03	0.000	0.17	0.03	0.000	0.06	0.01	0.000
**Member of Working Group**	0.11	0.03	0.001	0.02	0.03	0.573	0.05	0.04	0.001
**Constant**	2.76	0.13	0.000	-0.26	0.15	0.091			
**N**		1,109			1,109				
**Adj. R**^**2**^		0.44			0.34				
**F**		88.91			58.25				

Note: Standardized coefficients (ß) and standard errors (SE) are rounded to the second decimal place, p-values are rounded to the third decimal place. Missing values replaced full information maximum likelihood estimation.

Moreover, objective performance indicators were significantly related to research self-efficacy and academic career aspirations. Doctoral graduates who received a grade of summa cum laude had significantly higher research self-efficacy and academic career intentions. Also graduates with the grade of magna cum laude had higher research self-efficacy beliefs, which were indirectly related to higher academic career intentions. The same applied to first author article publications, which were positively and significantly related to research self-efficacy, and thereby indirectly to academic career aspirations. A small negative direct effect of the duration of the doctorate on research self-efficacy was observed and a negative indirect effect on academic career intentions. With respect to conferences, specifically contributions to international conferences were associated with higher self-efficacy and academic career intentions, directly and indirectly. A small positive effect of national conference contributions on research self-efficacy was observed. Whereas contributions to national conferences were positively related to academic career intentions via self-efficacy, a small negative direct effect was simultaneously observed. Moreover, having belonged to a working group within the doctorate was related to higher research self-efficacy and hence, indirectly to academic career intentions.

#### Gender, research self-efficacy and academic career intentions

To test for the hypothesized gender differences in research self-efficacy, a stepwise multivariate regression analyses was carried-out for medicine and life sciences separately, controlling for performance and experience (see Table 3). The results are consistent with the bivariate comparisons showing significantly lower self-efficacy beliefs of female medical doctoral graduates but with no gender differences in the life sciences. Hence, Hypothesis 4, which claims no gender differences in research self-efficacy when controlling for performance, is rejected for the field of medicine. In addition to the previous results, having conducted an experimental dissertation was significantly and positively related to research self-efficacy for the medical group. Moreover, having been a part of a working group during the doctorate was significantly and positively related to research self-efficacy only within the group of medical doctoral graduates. However, the duration of the doctorate was significantly and negatively related to levels of expressed self-efficacy for medical graduates only.

**Table 3:** Gender differences in research self-efficacy beliefs, multivariate regression analyses by field of study.

Independent Variables	Medicine			Life Sciences		
	ß	SE	p	ß	SE	p
**Female**	-0.16	0.04	0.000	0.02	0.04	0.593
**Summa cum laude**	0.09	0.04	0.043	0.27	0.08	0.001
**Magna cum laude**	0.04	0.05	0.383	0.13	0.08	0.115
**No grade**	-0.04	0.04	0.326	0.17	0.06	0.006
**(reference category: cum laude and lower)**						
**Publications: 1st Author**	0.27	0.04	0.000	0.23	0.05	0.000
**Publications: Co-Author**	0.00	0.04	0.995	0.01	0.05	0.817
**Duration of Doctorate**	-0.13	0.04	0.001	-0.02	0.05	0.601
**Experimental Dissertation**	0.10	0.05	0.029			
**National Conferences**	0.07	0.05	0.169	0.08	0.05	0.112
**International Conferences**	0.20	0.05	0.000	0.10	0.05	0.026
**Member of Working Group**	0.11	0.04	0.012	-0.06	0.06	0.257
**Constant**	2.61	0.15	0.000	4.45	0.33	0.000
**N**	571			538		
**Adj. R**^**2**^	0.35			0.16		
**F**	28.58			10.49		

Note: Standardized coefficients (ß) and standard errors (SE) are rounded to the second decimal place, p-values are rounded to the third decimal place. Missing values replaced full information maximum likelihood estimation.

In order to test Hypotheses 4 and 5, stepwise multivariate regression analyses were carried-out with academic career aspirations as the dependent variable, controlling for objective performance and experience in the first step and integrating research self-efficacy as the independent variable in the second step (see Table 4). The results remained consistent with the bivariate comparisons: no gender differences with respect to aspiring to an academic research career were observed in the life sciences (Table 4, Model 1ls) whereas gender differences remained significant in medicine (Table 4, Model 1med). Female medical graduates had lower academic career aspirations, even when controlling for performance outcomes (Table 4, Model 1med), leading to a rejection of Hypothesis 5 for the field of medicine.

**Table 4:** Stepwise regression analyses by field of study and academic career aspirations as dependent variable.

Independent Variables	Medicine						Life Sciences					
	Model 1med			Model 2med			Model 1ls			Model 2ls		
	Std. B	SE	p	Std. B	SE	p	Std. B	SE	p	Std. B	SE	p
**Research Self-Efficacy**				0.46	0.04	0.000				0.32	0.04	0.000
**Female**	-0.10	0.04	0.010	-0.03	0.04	0.394	0.02	0.04	0.724	0.01	0.04	0.822
**Summa cum laude**	0.17	0.05	0.000	0.13	0.04	0.002	0.27	0.07	0.000	0.19	0.07	0.009
**Magna cum laude**	0.06	0.05	0.169	0.04	0.04	0.316	0.02	0.08	0.812	-0.01	0.07	0.859
**No grade**	0.01	0.04	0.811	0.03	0.04	0.372	0.00	0.06	0.951	-0.05	0.06	0.392
**(reference category: cum laude and lower)**												
**Publications: 1st Author**	0.26	0.05	0.000	0.13	0.04	0.002	0.09	0.05	0.077	0.02	0.05	0.653
**Publications: Co-Author**	-0.03	0.05	0.535	-0.03	0.04	0.447	0.09	0.05	0.058	0.08	0.05	0.071
**Duration of Doctorate**	-0.11	0.04	0.007	-0.05	0.04	0.209	-0.02	0.05	0.713	0.00	0.05	0.943
**Experimental Dissertation**	0.15	0.05	0.002	0.11	0.04	0.011						
**National Conferences**	-0.06	0.05	0.248	-0.10	0.05	0.043	-0.06	0.05	0.170	-0.09	0.04	0.049
**International Conferences**	0.23	0.05	0.000	0.15	0.05	0.003	0.21	0.05	0.000	0.18	0.04	0.000
**Member of Working Group**	0.02	0.04	0.607	-0.03	0.04	0.466	0.03	0.06	0.593	0.05	0.05	0.391
**Constant**	1.51	0.14	0.000	0.30	0.17	0.076	1.24	0.28	0.000	-0.19	0.33	0.566
**N**	571			571			538			538		
**Adj. R**^**2**^	0.29			0.43			0.17			0.26		
**F**	20.76			35.08			9.79			15,37		

Note: Standardized coefficients (ß) and standard errors (SE) are rounded to the second decimal place, p-values are rounded to the third decimal place. Missing values replaced full information maximum likelihood estimation.

When controlling for research self-efficacy, gender differences in medicine disappeared (Table 4, M2med). Moreover, in support of Hypothesis 1, research self-efficacy significantly correlated with academic career intentions (Table 4, Models 2med and 2ls). In addition to the previous results, having conducted an experimental dissertation was positively related to academic career intentions in medicine. Furthermore, in medicine, first author publications were significantly related to academic career intentions. In accordance with the results from the path analysis, national conference contributions were negatively related to academic career intentions, although this effect was small but apparent in both medicine and life sciences (Model 2med, Model 2ls).

## Discussion

The goal of the current study was to explore the relationship between research self-efficacy beliefs on the intention to pursue an academic research career in the fields of medicine and basic life sciences, specifically addressing disciplinary and gender differences. An additional focus was laid on the control of objective scientific performance variables, which were neglected by several previous studies [31, 32, 52] and could have explained their reported gender differences in research self-efficacy [32]. The conducted analyses support that research self-efficacy beliefs are related to the intention to pursue a career in academic research, also when controlling for objective scientific performance. As hypothesized, medical doctoral graduates expressed lower research self-efficacy and academic career intentions. Lower academic career intentions were further statistically mediated by research self-efficacy beliefs: under control of research self-efficacy, medical doctoral graduates had even higher academic career intentions.

In addition, several indicators of objective scientific performance were identified as possible sources of research self-efficacy: the grade of the doctorate and first author publications were positively and significantly related to self-efficacy beliefs in both medicine and basic life sciences. Having been part of a working group (positive) and the duration of the doctorate (negative) were only significantly related to research self-efficacy within the group of medical doctoral graduates. Due to the fact that medical students in Germany usually start their doctorate as undergraduates [25], they tend to have less research experience in comparison to other PhDs students. This could explain why medical doctoral students exhibited a higher benefit from participating in a working group. Furthermore, being a part of a working group was the case for the majority of basic life sciences doctoral graduates but only for a small fraction of medical doctoral graduates. Moreover, medical doctoral students may interpret the time until finishing the dissertation as more meaningful, since medical dissertations are usually conducted within a shorter period of time [68].

International and national conference contributions were positively related to research self-efficacy and, thereby, had an indirect positive effect on academic career intention. However, contributions to national conferences had a direct negative effect on academic career intention. While this effect was comparably small, it is possible that graduates who contribute to national conferences meet more young researchers of their peer group and, as a result, are more aware of the competition in academic research. Another possible interpretation is that contributions to national conferences indeed offer an opportunity to practice ones’ presentation skills, but may not be understood as conducive in comparison to international conferences.

Moreover, some indicators of objective scientific performance were directly related to academic career intentions: a grade of summa cum laude (both fields) and first author publications (only medicine). The direct relationship may be explained by higher objective chances of academic career success. The result of first author publications only being significantly related to academic career intentions in medicine could be explained by higher standards for dissertations in the basic life sciences. In our sample, the majority of basic life sciences doctoral graduates had achieved at least one publication as a first author. In medicine, a publication as a first author was rarer and, therefore, may be interpreted as a more meaningful achievement. Another possible explanation is that only medical doctoral students with a specifically high research interest publish as first author.

Gender differences were apparent on the objective scientific performance level for both analyzed fields: women had fewer publications and attained lower grades. However, female life scientists did not express lower research self-efficacy beliefs or intentions to pursue an academic research career. This result differs from the study on biomedical PhDs by Gibbs et al. [18], who found significant gender differences in their sample. Since participants were asked retrospectively about their research self-efficacy and career intentions, these results may have been influenced by cognitive dissonance: participants who did not pursue an academic research career (more women) may have recalled their research self-efficacy beliefs as lower [73]. In contrast to the basic life sciences, female medical graduates expressed lower research self-efficacy and academic career intentions in comparison to their male counterparts. These differences were not explained by objective scientific performance. While research self-efficacy statistically mediated the gender effect on academic career intentions in the group of medical doctoral graduates, the difference in research self-efficacy remains to be explained.

Although a multitude of scientific performance and experience variables were controlled for, it is still conceivable that the observed gender differences are a result of unobserved heterogeneity. For instance, gender differences in research experience before the doctorate or types of publications (e.g. original articles vs. other types) were not assessed here. A further limitation of our results is that different experiences of supervision and mentorship and the anticipation of difficulties to combine an academic career with family duties were not measured. Relationships with supervisors and mentors might be another source of research self-efficacy and academic career intention. Empirical evidence suggests that females within the medical fields receive less mentorship [74, 75]. Both differing experiences with supervisory support and experiences with other researchers might contribute to gender differences. In addition, physician-scientists in clinical fields have to regularly combine patient care, research and teaching [76, 77]. While combining family life with an academic research career is not easy in general, it might be specifically challenging in academic medicine. Physician-scientists in Germany often conduct their research after their regular work day and even on week-ends [78, 79]. The fact that women, including female physicians, still carry the primary responsibility of childcare and domestic work [78, 80, 81], must render the challenge of combining family and a research career even more difficult for them. Researching in the evenings and on weekends might not be possible for them. Within a survey of physicians working in a university hospital in Germany, more female than male physicians indicated to have postponed child bearing due to work [78]. This could be a hint that females who anticipate these difficulties and don’t want to postpone family planning dismiss academic careers early.

Since the current study only analyzed a sample of doctoral graduates shortly after attaining their doctoral degree, the longitudinal development of research self-efficacy was not addressed. As a result of different levels of family duties or work experiences, research self-efficacy beliefs may evolve differently for male and female researchers in their ongoing careers. Accordingly, results of Abele [82] show no gender differences in vocational self-efficacy in medical graduates after the second state examination. However, after three years of work experience females expressed significantly lower vocational self-efficacy beliefs. These issues should be considered in future studies.

Another interesting question is whether there are differences in the pursuit of academic research between medical specialties. For instance, it is conceivable that physicians in fields with more clinical duties might be more likely to drop out of academic research than those in fields with lower patient contact. It is also possible that physicians wanting to pursue a career in academic research self-select themselves into fields in which the load of patient care is minimized precisely so that they can dedicate more time to research.

A methodological limitation of our study is its cross-sectional design; therefore, the results need to be interpreted cautiously with respect to causality and causal direction. It may also be possible that graduates with high academic career intentions indicate higher research self-efficacy to avoid cognitive dissonance. However, the vast empirical evidence (from experimental and survey research) on self-efficacy effects on achievement motivation and goal-setting, suggest that the correlations found within this study are not completely attributable to a reversed causality. While our measures of research self-efficacy were built upon already existing instruments, the instrument should be further validated with different samples of doctoral graduates.

The results of this study suggest that research self-efficacy is an important predictor of academic career intentions for recent PhD graduates. Future studies should analyze to what extent these are able to explain career decisions in later career stages. The effect of research self-efficacy on academic career intentions could vary per one’s career stage and experienced scientists may be more aware of actual career prospects in academic research (e.g. difficulties in keeping employed in academic research). In this regard, it is important to keep in mind that career intentions are necessary to follow a certain career path, but the extent to which intentions can be realized is limited by opportunity structures. As mentioned before, physicians face very good career opportunities in clinical careers. When the threefold threat of academic medicine is too demanding, physician scientists have an “easy way out”. Creating the framework conditions for a better balance between patient care and research might be an important step toward keeping physician scientists in research. Another factor that might turn physicians away from academia in Germany is remuneration. While clinically working physicians receive comparatively high salaries, those who focus solely on research receive the same remuneration as researchers from other fields, which is significantly lower [25, 79]. However, since most physicians in university hospitals combine patient care and research, it is unclear how often research is in fact related to a salary penalty in this specific context. While in the long run, continuing research is also an advantage for climbing the clinical career ladder [83], medical graduates may dismiss academia early due to higher opportunity costs in the first career stages [25]. Especially in the case of high educational debt and/or providing for a family, higher entrance salaries outside the university hospital may turn medical graduates away from research. The possible link between social background, salary and career choice should be addressed by future research.

## Conclusions and implications

The results of the study suggest that research self-efficacy beliefs are an important predictor of academic career intentions and, further, that medical doctoral graduates have a lower interest in academic research careers in comparison to their counterparts from basic life sciences. These differences were statistically explained by medical graduates’ lower research self-efficacy beliefs. As such, the study results suggest that the teaching of research related skills and knowledge in German medical education might not suffice to prepare medical students for their doctorate. This is in line with the recent critique of research content insufficiency in the current medical curriculum [84] and could be one reason why medical doctoral graduates express lower research self-efficacy beliefs in comparison to other life sciences doctoral graduates. Hence, our results point to the importance of integrating more research content into undergraduate medical studies. An increase in research content may lead to higher research self-efficacy beliefs [85], induce a higher research interest, and, as demonstrated by our results, induce interest in academic research careers.

Our results further suggest that academic career intentions shortly after the PhD do not explain the higher female dropout from academic careers in basic life sciences. In contrast, female medical graduates expressed lower interest in pursuing a career in academic medicine and this difference was no longer significant when controlling for research self-efficacy. As the low amount of female physician scientists is of international concern, an explanation for their lower research self-efficacy beliefs is an important topic for future studies to analyze. Such results may be important for future policy recommendations with regard to fostering the new generation of researchers.

In the current study, the separate analysis of gender differences for the fields of medicine and basic life sciences revealed that gender may not have an equivalent impact in different disciplines—even in related disciplines with a similar gender distribution. Many studies analyze various disciplines collectively, which may conceal important differences. Therefore, we suggest that previous studies re-analyze their data (when sufficient sample sizes are available) and that future research proceeds with differentiating the analyses per individual disciplines.

## Supporting information

S1 Appendix (DOCX)
