# Supplementary material for: Academic career intentions in the life sciences: Can research self-efficacy beliefs explain low numbers of aspiring physician and female scientists?
Source: PLoS One. 2017 Sep 14;12(9):e0184543. doi: 10.1371/journal.pone.0184543 (PMC5598975; doi:10.1371/journal.pone.0184543)
Supplement: S1 Appendix — (DOCX) [file pone.0184543.s001.docx]

**S1 Appendix**

Wording of Items of the Research Self-Efficacy Scale

| **Now that I have my PhD, I am sure I can…** |
| --- |
| …determine research strategies and research priorities for the future |
| …build up collaborations with other scientists |
| …raise third-party funds for research projects |
| …mount a research project (doctrine, formulating and handing in a research project) |
| …supervise and conduct long-term research projects  …frequently publish research findings in journals with peer review process |
| …compose a habilitation dissertation or several publications for a collective habilitation* |
| …get scientific recognition in the scientific community |
| …present my research at conferences |

Note: original scale in German, items translated. *The “habilitation dissertation” is a second academic thesis that succeeds the PhD and is common in Germany, Switzerland and Austria. The habilitation was for a period of time the only path to a position as full professor. Other options to qualify for professorship, such as junior professorships and leading a junior research group, are getting more popular in some fields and may slowly replace the habilitation in the future [1].

1. Konsortium Bundesbericht Wissenschaftlicher Nachwuchs. Statistische Daten und Forschungsbefunde zu Promovierenden und Promovierten in Deutschland. Bielefeld: Bertelsmann; 2013.
